# Allostatic Load and Preterm Birth

**DOI:** 10.3390/ijms161226209

**Published:** 2015-12-15

**Authors:** David M. Olson, Emily M. Severson, Barbara S. E. Verstraeten, Jane W. Y. Ng, J. Keiko McCreary, Gerlinde A. S. Metz

**Affiliations:** 1 Departments of Obstetrics and Gynecology, Pediatrics and Physiology, University of Alberta, Edmonton, AB T6G 2S2, Canada; eseverso@ualberta.ca (E.M.S.); verstrae@ualberta.ca (B.S.E.V.); jwng@ualberta.ca (J.W.Y.N.); 2 Canadian Centre for Behavioural Neuroscience, University of Lethbridge, Lethbridge, AB T1K 3M4, Canada; keiko.mccreary2@uleth.ca (J.K.M.); gerlinde.metz@uleth.ca (G.A.S.M.)

**Keywords:** allostatic load, allostasis, chronic stress, preterm birth, inflammation, two hits, multiple hit hypothesis, adverse pregnancy outcomes

## Abstract

Preterm birth is a universal health problem that is one of the largest unmet medical needs contributing to the global burden of disease. Adding to its complexity is that there are no means to predict who is at risk when pregnancy begins or when women will actually deliver. Until these problems are addressed, there will be no interventions to reduce the risk because those who should be treated will not be known. Considerable evidence now exists that chronic life, generational or accumulated stress is a risk factor for preterm delivery in animal models and in women. This wear and tear on the body and mind is called allostatic load. This review explores the evidence that chronic stress contributes to preterm birth and other adverse pregnancy outcomes in animal and human studies. It explores how allostatic load can be used to, firstly, model stress and preterm birth in animal models and, secondly, how it can be used to develop a predictive model to assess relative risk among women in early pregnancy. Once care providers know who is in the highest risk group, interventions can be developed and applied to mitigate their risk.

## 1. Introduction

**Figure 1:**
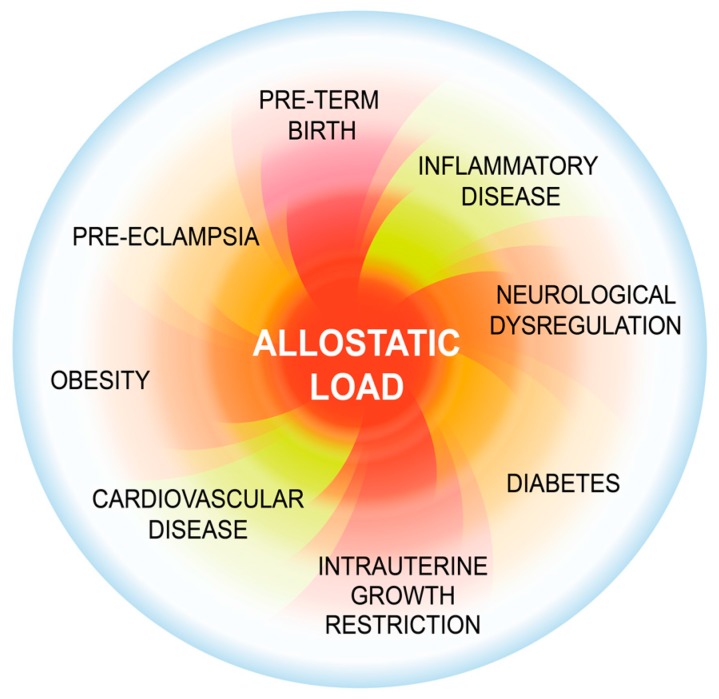
Allostatic load (AL) increases the risk for several perinatal and adult disease processes. Allostatic load comprises the wear and tear of stress over a lifetime on the body [1,2]. In chronic stress, the allostatic load increases as the body attempts to cope with stressors. When resiliency is overcome, inflammatory processes and neuroendocrine mediators, (e.g., cortisol and epinephrine) that normally maintain homeostasis now have a negative effect on the body, resulting in increased risk for numerous AL disease processes including preterm birth (PTB).

Preterm birth (PTB) is a complex perinatal period health issue that remains one of the greatest unmet medical challenges. The worldwide rate of PTB is >10% and increasing. Here, we develop a novel conceptual framework implicating the understudied phenomenon of allostatic load (AL). AL represents the wear and tear of stress on the mind and body and is both a risk and causal factor for PTB and possibly other adverse pregnancy outcomes (Figure 1). Its effects can be passed on through generations leading to poor pregnancy outcomes and adverse developmental trajectories. In this review, we will link the concept of allostatic load with PTB, describe animal models that generate both allostatic load and PTB for the study of the problem, and propose a process for developing a tool based upon allostatic load to stratify women into low and high risk groups for PTB.

## 2. Background

Preterm birth, which is associated with an intrauterine pro-inflammatory state [3], represents the leading cause of neonatal morbidity and mortality and is one of the most critical causal factors for disease in later life. For example, infants born preterm (*i.e.*, earlier than 37 completed weeks of gestation) are at greater risk of dying before the age of five and for developmental delay and other adverse health conditions including cardiovascular and metabolic diseases than infants born at term [4]. In spite of focused research efforts, the causes of PTB remain unknown in more than 50% of cases [5]. Both severe maternal distress during pregnancy [6,7] or pre-conceptual factors [8] have been associated with PTB.

### 2.1. Scope of the Issue

Many reports suggest that adverse perinatal programming by stress may increase the risk of PTB and low birth weight [6,7,9]. Prenatal exposure to endocrine disruptors in female rats [10,11] or to maternal undernutrition in humans [12] has been associated with increased metabolic and endocrine disease risk in the offspring. Gestational stress may affect levels of hormones and neuropeptides, including prolactin, progesterone, and oxytocin, which are involved in pregnancy maintenance and timing of delivery [13]. In animal studies, adverse experience was suggested to compromise the continuation of gestation [14].

To date, there is no standard method to measure AL in association with PTB. Typically AL assessment is categorized by physiological domains, beginning with the primary neuroendocrine mediators, then the secondary outcome domains including immune, metabolic, cardiovascular, respiratory and anthropometric, and finally the tertiary outcome domains [15]. Two or three elements are measured within each domain to determine a relative score for that domain. Biomarkers within these domains are usually systolic and diastolic blood pressure (SBP, DBP), body mass index (BMI), serum levels of cortisol, total cholesterol, triglycerides, glucose [16,17], fibrinogen and white blood cell count [18]. Other common biomarkers include pro-inflammatory cytokines, especially interleukin (IL)-6 [19], and C-reactive protein. More recently, telomere length was suggested as a biomarker of allostatic load as it demonstrates cellular aging [20]. Following the evaluation, the results are cumulated and ranked on various scales, which then result in an AL score [21].

Though these markers are important in defining allostatic load, they do not gauge social factors that have been identified as risk factors for PTB. These social markers include socioeconomic status, past traumatic events, marital or relationship issues, abuse, discrimination, loss of a close friend or family member and natural disasters. They demonstrate a clear indication of the type and extent of the stressors that the patient may be experiencing. Many of these factors can be measured through a variety of tests such as the Adverse Childhood Events (ACE) score [22] or our Wellbeing and Pregnancy Questionnaire [23]. Another determinant of AL is the epigenetic load of an individual that accumulates over time or experience [24]. The critical consideration for assessing PTB risk is to determine which domains and factors within domains provide the best estimate. In addition to the numerous ways to measure AL, there are at least a dozen methods to compute the composite AL score [25].

### 2.2. Our Working Construct

We propose that a composite AL score can predict risk for preterm birth and other adverse pregnancy outcomes and that this score should include environmental factors, epigenetic inheritance and the physiological indicators of AL. This derives because these factors are inseparable and reinforce one another. The following sections examine the evidence that supports this construct.

## 3. Evidence to Support Construct

### 3.1. Previous Attempts to Link Allostatic Load with Pregnancy and Birth Outcomes

Several researchers have quantified AL preconception, postpartum and during pregnancy using an index based on a count of regulatory biomarkers, determined by data availability. Some of these studies found no evidence of a relationship between maternal preconception AL and adverse pregnancy outcomes, including PTB or low birth weight [18,26], while others found women with a history of giving birth to low birth weight infants or PTB had a higher AL [19] (Table 1). Morrison *et al.* [27] attempted to determine whether this method of measuring AL during pregnancy was meaningful. They assessed the distributions of 10 biomarkers in pregnant and non-pregnant women and found a significant difference in the distribution of each AL-related biomarker. Among non-pregnant women, the high AL findings were consistent with previous studies (e.g., higher AL in women who are black, are older, and who have lower incomes). However, these associations were not present in pregnant women. The authors concluded that their approach to measuring AL may not provide meaningful information about chronic stress in pregnant women, where AL may reflect proximal factors in pregnancy more strongly than they represent exposure to chronic stress over a women’s lifetime.

**Table 1:** Methods used to assess allostatic load in association with pregnancy and birth outcomes.

Study	Data Source	Population	Data Collection	Biomarkers	Allostatic Load Scoring	Outcome
Wallace *et al*., 2013 [26]	Bogalusa Heart Study	African-American women	Preconception	SBP DBP Total cholesterol Triglycerides Glucose Insulin BMI Fibrinogen WBC	Contribution of each biomarker value to AL index weighted by loadings on the first principal component. This linear AL index is split into quartiles for analysis. Higher quartiles represent greater AL.	No evidence of a relationship between maternal preconception allostatic load and preterm birth or low birth weight infants.
Wallace *et al.*, 2013[18]	Bogalusa Heart Study	African-American women	Preconception	SBP DBP Total cholesterol HDL LDL Triglycerides Glucose Insulin Waist circumference	Score of 1 or 0 based on whether biomarker within high risk percentile or below based on data sample’s distribution, respectively. Score is summed for each biomarker to obtain AL score ranging from 0 to 9, which larger score indicated higher AL.	No evidence of a relationship between maternal preconception allostatic load and preterm birth or low birth weight infants.
Wallace and Harville, 2013 [28]	Tulane-Lakeside Hospital Department of Obstetrics and Gynecology	White or African-American	Pregnant: 26–28 weeks gestation	Cholesterol HbA1c DHEA-S Cortisol SBP	Uses z-score for each biomarker based on the data sample’s distribution. AL score for each subject is the sum of z-scores. Higher scores presents higher AL.	Gestational age decreased significantly with increasing allostatic load.
Morrison *et al*., 2013 [27]	NHANES 1999–2006	Civilian noninstitutionalized US population	Pregnant and non-pregnant	SBP DBP 60-s pulse rate Total cholesterol HDL-C CRP Albumin Creatinine HbA1c Homocysteine	Score of 1 or 0 based on whether biomarker within high-risk percentile or below based on data sample’s distribution, respectively. Score is summed for each biomarker to obtain AL score ranging from 0 to 10, which larger score indicated higher AL.	AL may reflect proximal factors in pregnancy more strongly than they represent exposure to chronic stress over a woman’s lifetime.
Hux *et al*., 2014 [19]	NHANES 1999–2006	Civilian noninstitutionalized US population	History of low birth weight infants and those who were preterm	SBP DBP Total cholesterol HDL HbA1c CRP BMI Albumin Creatinine	Score of 1 or 0 based on whether biomarker is within high risk percentile or below based on data sample’s distribution, respectively. Score is summed for each biomarker to obtain AL score ranging from 0 to 9, which larger score indicated higher AL.	Women with history of SGA or PTB had higher AL than did those with normal birth weight outcomes.

### 3.2. Chronic life Stress in Women

The cumulative effects of stress seem to be of particular importance to PTB risk [9]. Transgenerational and intergenerational stress may contribute to AL as a prognostic factor. Factors determining PTB risk may be passed on to the offspring through the maternal lineage, including repeated exposure to stress across generations [28]. Prenatal stress in the offspring, by permanently altering the inflammatory cytokine milieu, may predispose to pregnancy complications later in life [29]. Such programming of physiological and inflammatory responses in early life may transmit through subsequent generations [30]. Inter-generational programming of hypothalamic-pituitary-adrenal (HPA) axis activity during pre- and early postnatal development can become a key regulator of adult disease [31,32] and behaviour [33,34,35]. Thus, maternal stress during pregnancy may program physiological responses, PTB risk and birth outcomes across multiple generations.

We assessed chronic, lifelong stressors by designing the Wellbeing and Pregnancy Questionnaire and relating responses to spontaneous PTB by administering the questionnaire to 223 post-partum mothers who delivered preterm (75 cases) or at term (148 controls) [23]. Both individual and contextual variables that influence the stress response were examined. Several checklists designed for this study and previously validated research instruments were used to measure concepts related to stress and personal resources. Of all the separate instruments used to construct the questionnaire, only the Adverse Childhood Experiences (ACE) score [22] was significantly related to spontaneous PTB (*p* < 0.05), recording an odds ratio of 1.26 (95% CI 1.08–1.48). When this score was dichotomized into high (≥2 ACEs) *versus* low ACE it demonstrated that women with a high ACE score were over two times more likely to deliver preterm than those who had a score of 0 or 1 (Odds ratio 2.45; 95% CI 1.37–4.38, *p* < 0.05). Emotional and physical abuse as an adult were not associated with spontaneous PTB, but when calculating a combined childhood and adult abuse score, composed of the separate scores for childhood abuse, childhood neglect (from the ACE score) and adult emotional and physical abuse (from the Adult Assessment Screen [36]), we found a significant association with PTB (*p* < 0.05). A composite total stress score of all tools representing stressors or modifiers of the stress response was calculated as well, which appeared to be significantly related to the risk of spontaneous PTB (*p* < 0.05). When comparing a low to a high total stress score, the latter had an even larger OR than the total stress score itself (1.86 and 1.46 respectively). The presence of depressive symptoms during pregnancy was also significantly linked to PTB (*p* < 0.05). When examining more specifically the relationship between ACE score and spontaneous PTB, we found a direct relation between the number of ACEs experienced and PTB (χ^2^ test, *p* = 0.003). Furthermore, multivariate analysis showed that, after adjusting for maternal age, educational status, smoking and history of miscarriage, each additional increment of 1 on the abuse score scale for lifetime abuse increased by 34% the relative risk for having a spontaneous PTB. Combined, our findings suggest a close relationship between early life experiences and the PTB risk.

### 3.3. Animal Models

#### 3.3.1. Stress by Generations

**Figure 2:**
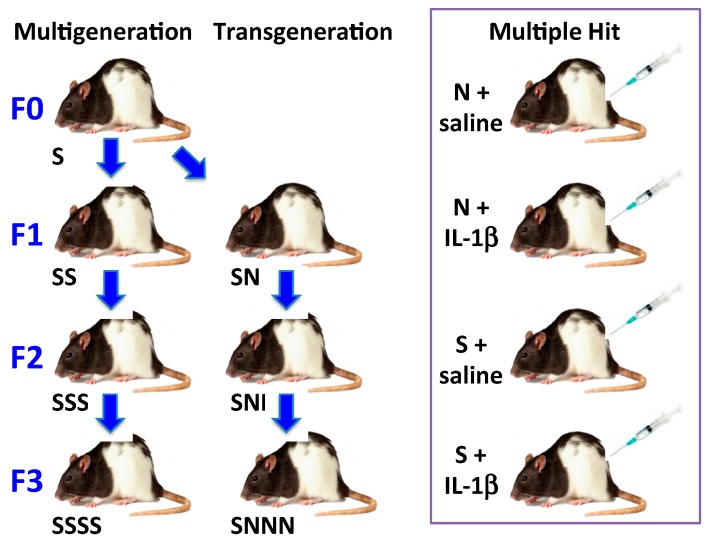
Allostatic load models in pregnant Long-Evans rats. We developed two longitudinal models, a multigenerational (**left**) and a transgenerational model (**center**), and a single generation (all F0) multiple hit model (**right**) that result in preterm birth plus other adverse pregnancy outcomes. In the multigenerational model, pregnant dams in each generation are subjected to restraint and swim stresses (S) on gestational day (GD) 12–18. The transgenerational model differs in that only pregnant dams from the F0 generation are stressed; their daughters and granddaughters are not stressed (N) during their pregnancies. In the multiple hit model, pregnant dams are stressed (S) or not (N) as in the longitudinal models plus they receive an intraperitoneal (IP) injection of saline or IL-1β (5 μg/kg) daily from GD 17 to delivery.

In order to test whether stress to each generation or to an ancestral generation affects pregnancy and developmental outcomes, we used pregnant Long-Evans rats to show that PTB risk, metabolic, endocrine and behavioural outcomes were affected by a single exposure to prenatal stress, which was generated by imposing gestational stress in the F0 parental generation (transgenerational stress) [24] or by stressing each generation (multigenerational stress) (Figure 2). Stressing each gestating generation produced a number of adverse pregnancy and newborn outcomes that accumulated with each succeeding generation. To our great interest we found the same results in the transgenerational model. The gestating F1 daughters and gestating F2 granddaughters became the generational longitudinal cohort or lineage. With each generation, prenatal stress (generated in the F0 generation in the transgenerational model) gradually reduced gestational length, maternal weight gain during pregnancy and maternal behavioural activity, and increased the risk of gestational diabetes in both models. Delayed offspring development was recognizable as early as postnatal day 7, with the greatest effect in the F3 offspring. Although we observed direct perinatal programming, we posit that indirect germ-line programming occurred in the F3 generation that were not exposed to stress unlike F1 (fetuses of F0) or F2 (primordial follicles in fetal ovaries of F1, present during F0 gestation) generations. We propose that gestational stress imposed on the great-maternal generation in our transgenerational model was passed on via the gametes to modulate gestational length, pregnancy outcomes and offspring health in the F3 generation. This finding demonstrates genuine transgenerational programming by stress.

#### 3.3.2. Multiple hit Model

**Figure 3:**
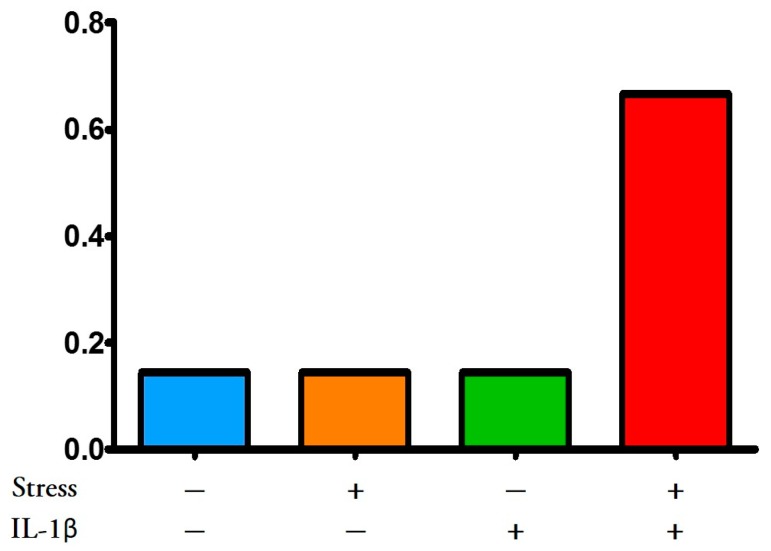
Adverse pregnancy outcomes in rats; frequency distribution. Two-hit hypothesis. Administration of stress (water swimming and restraint from gestational day (GD) 12–18) plus interleukin (IL)-1β (5 μg/kg, IP, GD 17-delivery, see Figure 2) to pregnant Long-Evans rats caused more pregnant dams to deliver early (most common outcome), deliver late or have other adverse pregnancy outcomes (dystocia or fetal resorption) than no treatment control, IL-1β or stress alone. These data support the two-hit allostatic load hypothesis [43].

While our intention was to develop a two-hit model, we had yet to solve the question of what the second hit should be. Previous literature suggested that male and female offspring of pregnant Long-Evans rat dams administered IL-1β from gestational days (GD) 17–21 (term = GD 22.5) had impaired cognitive performance (less time spent investigating a novel object) and female offspring demonstrated affective behaviour [37]. Maternal IL-1β administration also reduced utilization of progesterone in the hippocampus of female offspring as defined by conversion to dihydroprogesterone and allopregnanolone. Allopregnanolone plays an important role in the development of the central nervous system by promoting neuronal growth early in fetal life and later protecting neural development, promoting cognitive function, attenuating anxiety, and attenuating the oxytocin release apparatus [38,39]. Substituting restraint stress or chronically administering unpredictable stressors to pregnant dams for IL-1β administration also resulted in impaired cognitive function in offspring [37,40]. We therefore rationalized that both stress and IL-1β administered together might increase AL in pregnant dams and cause them to have more adverse pregnancy outcomes including PTB (Figure 2 multiple hit model). This PTB would occur in the F0 generation, which did not occur in the multigenerational F0 dams. We found that whereas neither stress alone in F0 (naïve) dams nor IL-1β alone had any effect on gestational length or other adverse pregnancy outcome, combining stress with supplementary IL-1β reduced gestational length (the most common outcome) and increased the number of other adverse pregnancy outcomes (Figure 3). Further, these two hits also caused poor health outcomes in the offspring, such as delayed brain development and altered connectivity leading to impaired behavioural performance [41]. Based upon these data, we propose a “two-hit” hypothesis whereby each significant “hit” by a stressful or inflammatory insult may cumulatively challenge the intricate mechanisms leading to parturition, thus increasing risk for preterm birth and other adverse pregnancy outcomes. This is derived from the concept first proposed by Knudsen [42] who showed that two gene mutations were necessary for retinoblastoma to occur in a patient. It can be imagined that women experiencing high allostatic load need only another insult or stress or “hit” to reach the tipping point that leads to an adverse pregnancy outcome. We hypothesize that more in-depth investigation into the women in our study [23] with two or more ACEs or life-long abuse, and supposedly already high ALs, would have revealed what these second hits may have been.

#### 3.3.3. Stress Hormones and Inflammatory Mediators Intersect at the Placenta: Potential Transgenerational Transmission Mechanism

**Figure 4:**
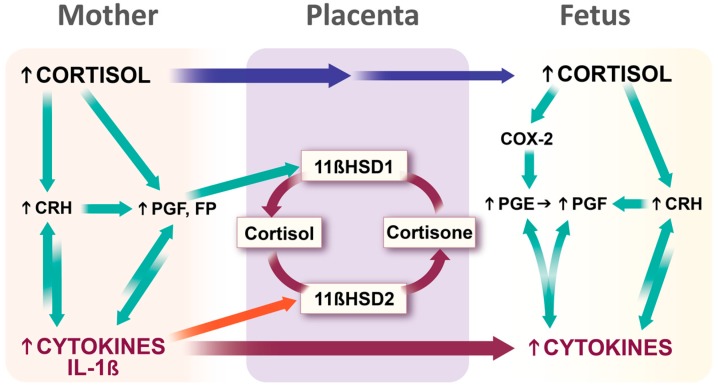
Role of placenta in transmission of generational stressors from mother to fetus. Increased fetal cortisol can be derived from elevated maternal cortisol concentrations via a high concentration gradient plus maternal cortisol-induced stimulation of corticotrophin releasing hormone (CRH) and prostaglandins (PGs) in placenta and fetal membranes. PGF2α in turn stimulates 11β-hydroxysteroid dehydrogenase type 1 (11βHSD1) that converts the less biologically active cortisone to cortisol. Further, CRH and PGF2α can stimulate cytokine production, and in turn cytokines stimulate CRH and PGF production. Several cytokines, including IL-1β, inhibit the enzyme, 11βHSD2, which converts cortisol to cortisone, thereby decreasing the metabolism of cortisol. The net effect of increased activity of 11βHSD1 and decreased 11βHSD2 activity is an increased cortisol gradient and reduced metabolism leading to higher concentrations of fetal cortisol. Maternal cytokines can stimulate the production of fetal cytokines directly through cytokine stimulation and indirectly through CRH and PGF2α. Increased cortisol and cytokines in the fetal compartment amplify the production of more fetal cytokines, PGs and CRH and permit more cortisol to cross the placenta thereby increasing fetal circulating cortisol concentrations. Legend: Green arrows stimulatory; red curved arrows inhibitory, straight maroon and blue arrows passage through placenta.

Exposure of a fetus to glucocorticoids (GCs) during maternal stress is regulated, in part, by the placental enzyme 11β-hydroxysteroid dehydrogenase types 1 and 2 (11βHSD1/2). Circulating levels of physiological GC are much higher in the maternal than fetal blood. This gradient is ensured by 11βHSD2 which catalyzes the rapid inactivation of GCs to their inert 11-keto forms, thus forming a natural barrier to maternal GCs [44,45]. Maternal stress downregulates the feto-placental 11βHSD2 gene [46] and thereby excess GC levels caused by severe maternal stress may pass the placenta to reach the fetus [47,48]. A number of groups including ours have found that inflammatory mediators, including interleukins (ILs), decrease activity of 11βHSD2 whereas prostaglandins (PGs) increase the activity or expression of its counterpart, 11βHSD1, which promotes metabolism of cortisone to active GC [3,49]. Excess GCs may stimulate fetal membrane PG production [50,51] which upregulates 11βHSD1, and PGs and cytokines are mutually stimulatory (Figure 4). Thus, excessive maternal GCs, cytokines or PGs can lead to 11βHSD2 inhibition/11βHSD1 activation thereby increasing fetal GC concentrations that modulate the developing fetal HPA axis and its regulation in later life, altering brain development and HPA axis functions throughout the life course [52] including changes in memory and behaviour [48]. In this way, it is possible that stress in one generation may affect the next leading to a number of adverse outcomes including several associated with pregnancy and development.

#### 3.3.4. Epigenetic Associations

Our striking phenotypic impairments in the F3 generation suggest a genuine ancestral epigenetic inheritance whereby the epigenetic modifications have been passed via the gametes whose epigenome escaped reprogramming [53,54]. Indeed, phenotypic findings in behaviour and physiology were supported by molecular changes involving epigenetic regulation of gene expression whereby ancestral stress altered microRNA (miR or miRNA) expression patterns in brain and uterus of non-pregnant, lactating F2 mothers. In particular, stress led to upregulation of miR-200b and downregulation of miR-429, which may modulate gestational length. When upregulated, miR-200b may suppress transcription factors *Stat5b*, *Zeb1* and *Zeb2* mRNA levels in the lineage of pregnant dams exposed to prenatal stress. Furthermore, stress also increased miR-181a expression in the placenta. miR-181a has been associated with PTB in humans and may serve as a marker of shortened gestation [36]. These and other observations suggest that stress leads to miRNA changes in brain [55], uterus and placenta [24] and that the mechanisms involved in the timing of parturition and associated behavioural and physiological signatures may be programmed through the maternal lineage. The identification of epigenetic signatures of PTB in clinically accessible tissues, particularly maternal leukocytes but possibly placenta, offers the potential for predictive and preventive studies related to poor pregnancy outcomes.

The mechanism for transgenerational inheritance of epigenetic information from mother to offspring remains uncertain although strong evidence exists for it (extensively reviewed in Babenko *et al.* [56]). Recent discoveries indicate that the placenta releases miRNAs, either directly into the maternal and fetal circulation or encapsulated into exosomes, and that maternal miRNAs can pass through the placenta to the fetus [49,50,51]. This could be another method of transgenerational transfer of stress effectors from mother to fetus as well as possible predictive biomarkers.

#### 3.3.5. Stress, Inflammatory Mediators and Preterm Birth

**Figure 5:**
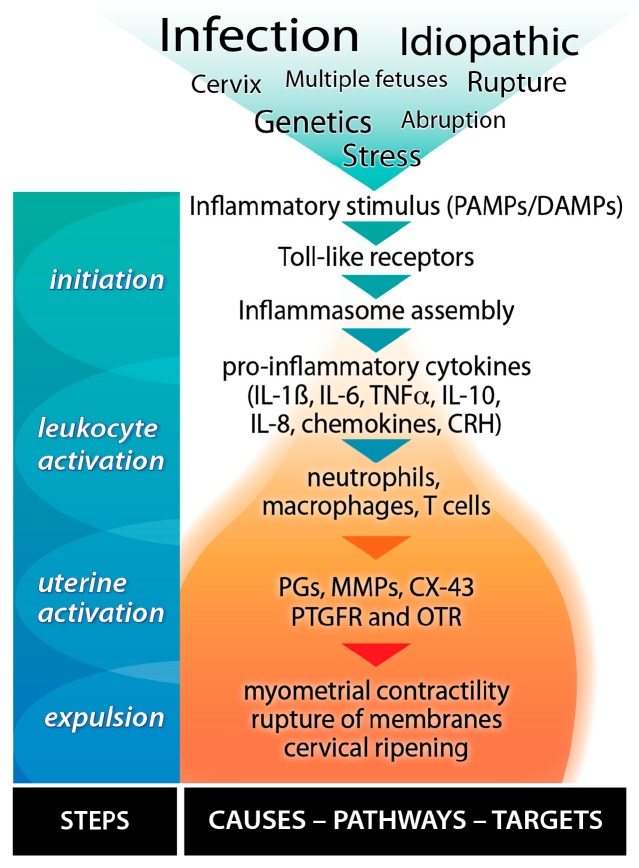
Inflammatory cascade leading to preterm birth. Stress, infection and other initiators stimulate Toll-like receptors (TLRs) to produce cytokines and chemokines that stimulate peripheral leukocytes to infiltrate the uterus. In turn, this leads to further stimulation of cytokines and chemokines plus the production of prostaglandins (PGs), matrix metalloproteinases (MMPs), and uterine activation proteins such as connexin-43 (CX-43), the PGF receptor (PTGFR) and the oxytocin receptor (OTR). The expression of these and many other proteins transform the uterus of pregnancy to the uterus of delivery via stimulating the physiological changes in myometrial contractility, membrane rupture and cervical ripening.

We have developed, tested and confirmed a robust pathway scheme that leads from several different causes of PTB to the transformation of the uterus from pregnancy to active labour (Figure 5) based upon the literature and our own experiments. Parturition is the transition from the pro-pregnancy and anti-inflammatory state to the pro-labour, pro-inflammatory state. Inflammatory processes play significant roles in most, if not all, labours, regardless of the presence of infection, etiology, or timing of delivery [57,58]. Inflammatory processes activate parturition and are intertwined with early (upstream), mid (midstream), and late (downstream) events in the birth cascade. Toll-like receptors (TLRs) are identified as upstream mediators of inflammatory cytokine and chemokine synthesis in both infection-induced PTB and normal delivery [59]. A receptor family that binds pathogen-associated molecular patterns (PAMPs) or damage-associated pathogens (DAMPs), the TLRs are expressed in the maternal decidua and during gestation in placental trophoblasts and membranes [59,60]. Upon activation they elicit pro-inflammatory cytokine and chemokine expression (e.g., IL-1β, IL-6, *etc.*), which are early mediators of the inflammatory response. Inflammatory cytokines activate and attract leukocytes from the peripheral blood into the uterus [61]. As more leukocytes invade the uterus, midstream events, including expression changes in uterine activation proteins (UAPs) and cellular adhesion molecules (CAMs) occur. The latter bind and tether the infiltrating leukocytes to create an inflammatory microenvironment [62] in which the inflammatory response is amplified through the release of pro-inflammatory cytokines, corticotrophin releasing hormone (CRH), and PGs, causing further expression of UAPs, cytokines, chemokines, and PGs, thereby transforming the uterus of pregnancy to the uterus of labour [57].

Drawing together these diverse elements (multi- and transgenerational stress, multiple hits, inflammatory mediators, and uterine transformation for labour) and their individual and combined effects upon adverse pregnancy and developmental outcomes should hopefully provide new insights into mechanisms, means to diagnose risk and new therapies to treat them. We next explore allostasis and allostatic load as unifying concepts in this regard.

## 4. Allostasis and Allostatic Load

### 4.1. Definitions

Originally proposed in the late 1980s by Sterling and Eyer, allostasis is the adaptive process for actively maintaining stability through change [1]. Allostasis is derived from the Greek “*allo*”, meaning “variable”, while “*stasis*” means “stand”. It is the way in which the body maintains homeostasis when exposed to stressors. It involves the stress response along with the production of inflammatory cytokines, adrenal hormones and neurotransmitters that help us adapt to new situations and challenges [2,63,64]. The brain plays a central role in allostasis by controlling various mechanisms simultaneously. In acute situations, allostasis is beneficial for the body, as it is essential for the body to respond and adapt to stressors. However, when allostasis is prolonged—in chronic or repetitive stress (called “hits” according to the Knudson multiple hit hypothesis [42])—the autonomic nervous system and HPA axis are repetitively activated and the neuroendocrine (cortisol, epinephrine, norepinephrine, dopamine) and inflammatory adaptive processes can now create damage through precipitating diseases including PTB. For this, McEwen adapted the term “allostatic load”. [65], defined as the cumulative results of allostasis. In other words, it comprises the wear and tear of allostasis over a lifetime on the body and the brain [17]. Allostatic load can therefore increase inflammatory cells and cytokines in tissues and cause increased susceptibility to infection and inflammation and many other adverse health conditions [2,66,67].

### 4.2. Types of Stressors

**Figure 6:**
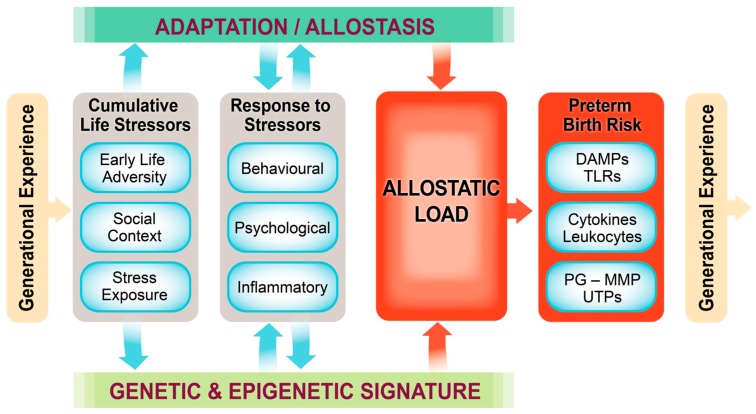
Conceptual framework linking transgenerational allostatic load and preterm birth. Building from the model of Saban *et al.* [69] and our own experimental data, we developed the Allostatic Load and Preterm Birth Conceptual Framework. This model provides a context for generational programming in connection with genetic and epigenetic signatures, multiple “hits” or stressors throughout the life course, downstream responses to these stressors leading to high allostatic load, and ultimately a sequence of distinct inflammatory and other mediator responses that lead to uterine activation, transformation and preterm birth (refer to Figure 5). The consequences of these effects are passed on to the next generation. See text for detailed description.

We propose categorizing stressors or hits into major groups, organized around transgenerational stressors, the genetic and epigenetic predisposition, early life stressors including *in utero* events, social context, life-long stress exposure, the response to stressors including behavioural, psychological and inflammatory, and also the epigenetic signature that results from these life experiences. Daskalakis [68] argues that within a given context vulnerability is enhanced with three hits and failure to cope with adversity accumulates. These three hits are the (epi)genetic predisposition existing at the time of conception (*i.e.*, the (epi)genetic inheritance from both parents), earlylife environment, including *in utero* and the first year of life, and later-life environment, both having a large epigenetic component. These susceptibilities, as suggested by Saban *et al.* [69], could lead to a pro-inflammatory epigenetic signature that bridges the psycho-social environment and tilts the individual towards disease vulnerability. We have integrated the concept of Saban *et al.* [69] and our own data into a conceptual framework that illustrates how multiple hits involving generational and life-course experience lead to allostatic load and PTB (Figure 6).

### 4.3. Our Conceptual Framework

Based upon concepts by Saban *et al.* [69], the Allostatic Load and Preterm Birth Conceptual Framework (Figure 6) asserts that an individual acquires generational experience through its genetic and epigenetic inheritance. This includes transgenerational inheritance via its germ cells or via placental transfer (e.g., of miRNA [70]) or by placental transfer of stress hormones and mediators (Figure 4). Early life adversity can occur *in utero* as well as in the newborn and early childhood periods, while social context and stress exposure are also major contributors to allostatic load. Allostasis is the response to these experiences including the behavioural, psychological and inflammatory. As the allostatic load exceeds the ability to cope, the risk for PTB or other adverse pregnancy or developmental outcomes increases likely through activation of inflammatory, epigenetic, hormonal and/or neurotransmitter pathways. In the case of PTB, these mechanisms include increased invasion of the uterus by leukocytes plus increased cytokine, chemokine, PG and matrix metalloproteinase output, stimulation of uterine activation gene expression and finally labour and delivery.

### 4.4. Experimental Models of Allostatic Load and PTB

Most studies and reviews of allostatic load and adverse health outcomes emphasize the longitudinal nature of accumulated lifetime or transgenerational stress [15,65,69,71]. In our studies, we observed increases in plasma corticosterone, the active glucocorticoid in the rat, with each generation in both transgenerational and multigenerational models. Increases in circulating glucocorticoids, neurotransmitters (e.g., dopamine, epinephrine or norepinephrine), and anti-inflammatory cytokines (e.g., interleukin-6) are described by Read and Grundy as the primary mediators of allostatic load [15]. Stress leading to increased neuroendocrine mediators sets off an accumulation of events leading to secondary outcomes including altered metabolism and metabolic indicators (e.g., insulin, glucose, total cholesterol, triglycerides, visceral fat depositing), cardiovascular indicators (e.g., systolic and diastolic blood pressure) and inflammatory proteins (e.g., C-reactive protein, fibrinogen). We observed hyperglycemia and decreased maternal weight—evidence of a metabolic disturbance likely due to gluconeogenesis—in both models. Tertiary outcomes are the result of the primary and secondary events and are the most easily observable health outcomes. In this model, they include changes in maternal behaviour and pregnancy outcomes. Read and Grundy observed the effects of allostatic load and health in an older population. They found that allostatic load predicted slower walking speed. Our observations extended further into the following generation and thus suggest an extension of their concept. We propose that the transgenerational passage of adverse health outcomes to the offspring should also be included among the tertiary outcomes. In this sense, we observed the tertiary outcomes of altered F0 maternal behaviours (decreased tail chasing and decreased exploratory activity) and delayed F1 offspring neurodevelopment (decreased negative geotaxis and reduced cortical neuronal density) plus changes in miRNAs, a marker of epigenetic change. Later in life, the F1 daughters in both the transgenerational and multigenerational models had an increased incidence of preterm delivery, and with each subsequent generation the primary, secondary and tertiary domains of allostatic load became more exaggerated [24,41]. In terms of human studies, our work in a cohort of Canadian women [23] demonstrated that adverse childhood experience prior to the age of 18 or life-long abuse are associated with PTB at an average age of 28.

However, the theory of acquiring allostatic load does not need to be restricted to life-long or transgenerational accumulation of stress. Allostatic load can also be achieved by an accumulation of stressors over a short duration of time. We demonstrated this using our multiple hit model (Figure 2 and Figure 3) whereby combining stress plus administration of the pro-inflammatory cytokine, IL-1β, led to PTB and other adverse pregnancy outcomes whereas neither stress nor IL-1β alone affected pregnancy outcomes in F0 rat dams. The multiple hit theory [72] has been used to explain epilepsy [73] and schizophrenia [74] among other diseases. Mor’s group exposed the mouse to a viral infection at the time of implantation, which increased the sensitivity of maternal immune cells to later bacterial infection and PTB [75].

While our data are limited at this writing, we have observed similar changes in primary, secondary and tertiary outcomes in our two-hit model as in our longitudinal generational models and hope soon to confirm that the multiple hit model mimics the longitudinal models. From the lessons learned regarding allostatic load markers for PTB in these animal models and the retrospective analysis of human data, our goal will be to develop a predictive model highly specific for assessing PTB risk in women during early pregnancy in order to identify those in the highest risk group who would be candidates for interventions to mitigate their risk.

### 4.5. Using Allostatic Load to Predict PTB Risk

Allostatic load markers have the potential to be incorporated into an effective risk model for PTB and other adverse pregnancy outcomes because they assess and integrate multiple systems. It is an index of multisystem physiologic risk that is used as a measure of the cumulative toll of physiologic and psychological stress [76]. It assumes multisystemic interactions and incorporates subclinical biomarkers of neuroendocrine, inflammatory, and metabolic function into a single index score [25]. Allostatic load is higher in marginalized populations and is associated with many adverse health outcomes; therefore, it may be a better predictor of adverse pregnancy outcome than traditional individual measures [18,65,77,78,79,80]. In the existing literature (e.g., Table 1) for allostatic load and PTB, two studies from the same group but using different non-specific markers of allostatic load before conception indicate there is no relationship between allostatic load and PTB [18]. But when the same authors used allostatic load markers more specific for PTB, they observed a significant relationship between elevated allostatic load and decreased gestation length [81]. In a retrospective study, specific allostatic load assessment had higher scores for women who delivered preterm than the term gestation reference group (*p* = 0.001); a similar outcome was noted when preeclampsia was the outcome measure [19,21]. Hence if the appropriate time points during pregnancy and allostatic load markers are selected, allostatic load has high potential to be an effective and relatively straightforward marker of PTB risk.

## 5. Developing an Allostatic Load Model for Predicting PTB Risk

We started searching for applications of allostatic load to predicting disease risk by searching PubMed (August 2015) for articles using the search term allostatic load. This generated 808 articles. We systematically scanned the titles and abstracts of these articles and chose those most relevant. Upon reviewing them, we established a working definition of allostatic load and its assessment. Next we narrowed the scope of our search to allostatic load and pregnancy. This search resulted in 27 papers that included one on preeclampsia and three on PTB. After reading each relevant article in detail, we narrowed the search to allostatic load and preterm birth, which generated 13 results, and incorporated the relevant articles. We then compared the PTB literature to other health problems involving allostatic load including preeclampsia, cancer and multiple psychological disorders [82,83].

We will use our conceptual framework of allostatic load and PTB (Figure 6) as a guide and apply it to the process of concept analysis to build the predictive model. This is a tool first developed in mathematics [84,85] and then applied successfully in nursing science [86]. It relates concepts to observations through question formation to establish the defining characteristics of the theory. The observations can be literature examples or experimental results, which are called case studies. By iteratively linking questions with case studies, the model takes shape. Typically a conclusive model does not result from concept analysis, but instead an assumed conclusion is formed that leads to suggestions for further research and a refinement of the model [86]. Hence the working theoretical model that results is constantly evolving, improving with each iteration of the research cycle. The tool can be applied at any time for solving a complex health problem, but it will continue to evolve and improve.

We are just beginning to develop a prognostic test for PTB. Because of the ability of allostatic load to affect multiple systems, it can be hypothesized that it contributes to the pathogenesis of PTB and thus would be an appropriate tool to assess PTB risk. Future research into this area will need to ask (1) which markers of allostatic load predict PTB best; (2) whether allostatic load measured in pregnancy is associated with increased odds of delivering preterm; (3) how early in pregnancy or pre-conceptually can allostatic load be measured and associated with PTB risk; (4) whether tailored allostatic load models need to be developed for varying cultures, jurisdictions and environments; and (5) whether allostatic load models specific for preterm birth are better at predicting PTB than other adverse pregnancy outcomes such as preeclampsia or gestational diabetes [24,41,87,88].

## 6. Conclusions

The future of research in the field of women’s pregnancy health and perinatal health in general will be at the population and clinical level and the study of allostatic load will be a leader in this effort. But the way in which population and clinical health research are addressed will be quite different than in the past. First, at this time there is too little reliance upon animal models that inform about basic mechanisms when framing clinical and epidemiological questions. Animal models of allostatic load will provide new evidence upon which to frame questions in the human context. Secondly, instead of the retrospective approach, the workhorse of epidemiological inquiry, research at the population and clinical levels involving the principles of allostatic load, gleaned from basic studies, will be prospective in order to predict women at risk of adverse pregnancy outcomes. The process of concept analysis may permit fewer women to be recruited into studies while retaining adequate power due to its iterative nature. The bane of PTB prospective trials when it is not known who is at risk is the large number of subjects required. Concept analysis will be used in a constantly evolving, iterative fashion testing predictive concepts in human cohorts thereby developing the algorithms required to ultimately and as quickly as possible identify women in the highest risk groups so that they can receive appropriate care to mitigate their pregnancy risks.
